# HIV now: why human rights matter more than ever

**DOI:** 10.7448/IAS.16.1.18968

**Published:** 2013-12-10

**Authors:** Chris Beyrer

**Affiliations:** Department of Epidemiology, Johns Hopkins Bloomberg School of Public Health, Baltimore, MD, USA

We are living in an extraordinary moment in the HIV response. Advances in science, implementation, community engagement and political support have led to unprecedented optimism and measurable declines in new forms of HIV infections in many communities, populations and affected countries. There is growing realization that through strategic use of the prevention, treatment and care advances we now have in hand, the fourth decade of the AIDS pandemic could be where we finally achieve meaningful and sustained reductions in new HIV infections, close to full life expectancy for those living with the virus, and integration of HIV into health services, which could be beneficial to both HIV programmes and strapped health systems. We are learning how to use the powerful insight that effective early HIV treatment is a potent HIV-preventive intervention for sero-discordant couples and may have preventive impacts at community and population levels. And we have new and combined prevention tools and technologies (and more in the research and development pipeline), which could markedly increase prevention choices and offer real promise in protecting those most in need of prevention.

For many, this bright moment has come too late. By the end of 2013, AIDS will have claimed more than 35 million human lives and affected countless more. We must not lose our urgency or flag in this key stage of our shared struggle against this virus.

What could hold us back? What could give HIV, again, the edge over humanity's best efforts to control spread and maintain the health and well-being of those living with HIV? While antiretroviral drug resistance is a real concern and adherence to safer behaviours, condom use and prevention technologies are on-going challenges, these can and are being addressed and overcome. The epidemiologic data suggest that much more fundamental issues are at play where HIV rates are rising, not falling, and where countries are facing expanding epidemics. Stigma, discrimination, human rights abuses and the criminalization of people and communities, including people who use drugs, sexual and gender minorities and sex workers, continue to constitute our greatest barriers to control of the pandemic. Failed policies and flawed politics are at the heart of our collective failures in the HIV response. This must change, not simply because the realization of the rights and dignity we all share as human beings is fundamental to all of our lives, but because the limitations on these basic rights and freedoms continue to aid and abet the HIV pandemic.

HIV rates continue to rise across the vast eastern Europe and central Asian region, driven by policy failures in the provision of sterile injecting equipment, political refusals to provide evidence-based drug treatment (such as methadone and buprenorphine) and harsh punitive polices and practices toward sex workers and gay, bisexual and other men who have sex with men. In many of these states, including the largest, the Russian Federation, recent policies have become so repressive that it is becoming difficult to even assess the spread of HIV as new anti-homosexuality propaganda laws make it challenging to reach men in need of services.

The most impressive gains against the virus have arguably been made in the severe epidemics in east and southern Africa, where behaviour change and condom usage, prevention of mother-to-child transmission, male circumcision and, arguably, expanded treatment access have together shown real traction against HIV spread. But even where HIV rates have declined among reproductive aged adults, limitations on essential services continue for many – again, driven by exclusion and discrimination of those deemed unworthy. Sex workers are denied PMTCT services in Tanzania. African gay men with sexually transmitted infections cannot find providers who will treat them in safety and dignity. In too many countries, people who inject drugs find that their substance use history is used to deny them HIV treatment. Transgender persons are excluded from care, particularly transgender women, who are the most disproportionately burdened of any community at risk. And persons in detention and prison worldwide continue to suffer from lack of access to essential services. These exclusions are violations of the right to health and to dignity and, because untreated HIV infection is fatal, of the right to life itself. This is morally unacceptable and scientifically indefensible.

If we cannot begin to provide essential services to those who need them most, HIV will win. And compassion will lose. Humanity will lose. Because we will have let bigotry prevail – and let our Achilles heel, discrimination, undermine the magnificent achievements we have made in the HIV response.

UN Secretary – General Ban Ki-moon spoke about the need for inclusion and about the universality of human rights for sexual and gender minorities on Human Rights Day in 2012:

The very first article of the Universal Declaration of Human Rights proclaims that, ‘All human beings are born free and equal in dignity and rights.’ All human beings – not some, not most, but all. No one gets to decide who is entitled to human rights and who is not. Let me say this loud and clear: lesbian, gay, bisexual, and transgender people are entitled to the same rights as everyone else. They, too, are born free and equal.

The road to full inclusion of those in need will not be easy. Indeed, we are currently seeing dangerous signs of increasing discrimination, as in Russia, Uganda, Cameroon and numerous other countries – but this is an essential struggle for the next phase of the HIV response. The great TB researcher, George Comstock, used to say: “TB anywhere is TB everywhere.” He meant that TB could not be controlled if all who needed treatment were not reached. On Human Rights Day, we must recognize that HIV anywhere is HIV everywhere. And we must commit to all in need. No exceptions. That is what human dignity demands.

